# Study on Temperature and Synthetic Compensation of Piezo-Resistive Differential Pressure Sensors by Coupled Simulated Annealing and Simplex Optimized Kernel Extreme Learning Machine

**DOI:** 10.3390/s17040894

**Published:** 2017-04-19

**Authors:** Ji Li, Guoqing Hu, Yonghong Zhou, Chong Zou, Wei Peng, Jahangir Alam SM

**Affiliations:** 1 Department of Mechanical and Electrical Engineering, School of Aerospace Engineering, Xiamen University, Xiamen 361005, China; 19920130154215@stu.xmu.edu.cn; 2 Department of Mechatronics Engineering, School of Mechanical & Automotive Engineering, South China University of Technology, Guangzhou 510640, China; mepeng.wei@mail.scut.edu.cn (W.P.); mejahangir@scut.edu.cn (J.A.S.); 3 Fujian Wide Plus Precision Instruments Co. Ltd., Fuzhou 350015, China; zyh@wideplus.com (Y.Z.); c@wideplus.com (C.Z.)

**Keywords:** piezo-resistive pressure sensor, temperature compensation, static pressure effect, KELM, CSA, simplex

## Abstract

As a high performance-cost ratio solution for differential pressure measurement, piezo-resistive differential pressure sensors are widely used in engineering processes. However, their performance is severely affected by the environmental temperature and the static pressure applied to them. In order to modify the non-linear measuring characteristics of the piezo-resistive differential pressure sensor, compensation actions should synthetically consider these two aspects. Advantages such as nonlinear approximation capability, highly desirable generalization ability and computational efficiency make the kernel extreme learning machine (KELM) a practical approach for this critical task. Since the KELM model is intrinsically sensitive to the regularization parameter and the kernel parameter, a searching scheme combining the coupled simulated annealing (CSA) algorithm and the Nelder-Mead simplex algorithm is adopted to find an optimal KLEM parameter set. A calibration experiment at different working pressure levels was conducted within the temperature range to assess the proposed method. In comparison with other compensation models such as the back-propagation neural network (BP), radius basis neural network (RBF), particle swarm optimization optimized support vector machine (PSO-SVM), particle swarm optimization optimized least squares support vector machine (PSO-LSSVM) and extreme learning machine (ELM), the compensation results show that the presented compensation algorithm exhibits a more satisfactory performance with respect to temperature compensation and synthetic compensation problems.

## 1. Introduction

Due to the fact pressure usually plays an important role in many industrial processes, a widespread need for pressure sensors has become a reality. One of the most widely used pressure sensors, called piezo-resistive pressure sensor, is made of monocrystalline silicon and fabricated by standard Micro-Electro-Mechanical System (MEMS) craft which conforms to the piezo-resistive principle [1,2,3]. Since the manufacturing process of the pressure sensor is complicated, some temperature-sensitive defects including inconsistent doping concentration, mismatched thermal expansion coefficients of packaging materials and electronics performance may have a dramatic effect on the output if the environmental temperature varies, i.e., they have a temperature effect [4,5,6]. Besides the temperature effect, the static pressure effect is another key problem that should be taken into consideration when the variable to be measured is differential pressure. Even if the environmental temperature is stable, static pressure can also yield measurement errors in the asymmetric structure of the sensible part of the sensor. Consequently, the input–output characteristics of monocrystalline silicon piezo-resistive pressure sensors becomes non-linear when the environmental temperature or the static pressure change [7].

In order to eliminate the temperature effect in diverse measurement systems, lots of approaches have been proposed, which can be typically categorized into two groups: hardware compensation and software compensation. Hardware compensation modifies the measurement circuits by adding electronical components or develops new structures to achieve more satisfactory performance [8,9,10,11]. The complicated debugging process, high cost and relatively limited compensation precision has restricted the spread of hardware compensation applications in engineering practice.

The software compensation approach deems the temperature compensation as a regression problem. The conventional software compensation methods of look-up table, spline interpolation and surface fitting are based on mathematical computation [12,13,14,15]. The conventional software compensation approaches seem easy to implement, but when one notices that the compensation quality is proportional to the data scale and the ill-condition dilemma in solving the normalization equation, they become less attractive. On the other hand, their vigorous algorithm robustness and fault tolerance ability has led researchers to focus a great deal of attention on artificial intelligence compensation approaches. The most up-to-date software compensation methods are rooted in artificial intelligence include neural networks [16,17,18,19], support vector machines (SVM) and least squares support vector machine (LSSVM) [20,21,22]. The classic back-propagation neural networks may suffer from the dimensionality curse, local minima, under-fitting or over-fitting, etc. Vapnik [23] developed the SVM, the core of which is the structural risk minimum principle constructed by the empirical risk minimum principle and confidence intervals. Due to the merits of the structural risk minimum principle, the SVM is able to resolve classification and regression problems with relatively small samples. Aiming at alleviating the computational burden of SVM, Suykens [24] proposed the LSSVM, which converts the inequality constraints in traditional SVM into linear equations. Huang et al. [25] presented a fast learning single layer network called extreme learning machine (ELM), wherein the weights and biases of the input layer are randomly assigned within the range of (0, 1) and the weights of the output layer are calculated by a pseudo inverse operation. In combination of the regularization theory, kernel trick and the ELM, the kernel extreme learning machine (KELM) is not only a computationally efficient network, but also has an elegant generalization ability [26].

Although a lot of schemes have been put forward to tackle the issue of temperature compensation, approaches related to synthetic compensation of the differential pressure sensor which consider both temperature and static pressure are rarely reported. In an attempt to compensate the temperature error and static pressure error at the same time, the KELM is adopted in this research. The regularization parameter and the spread parameter of the kernel function govern the KELM’s actual regression precision, hence a parameter selection strategy like the coupled simulated annealing (CSA) hybrid Nelder-Mead simplex searching is employed. Finally, a well-trained synthetic compensation model for differential pressure measurement under different static pressure and temperature conditions is established.

## 2. Temperature Effect and Static Pressure Effect

**Figure 1:**
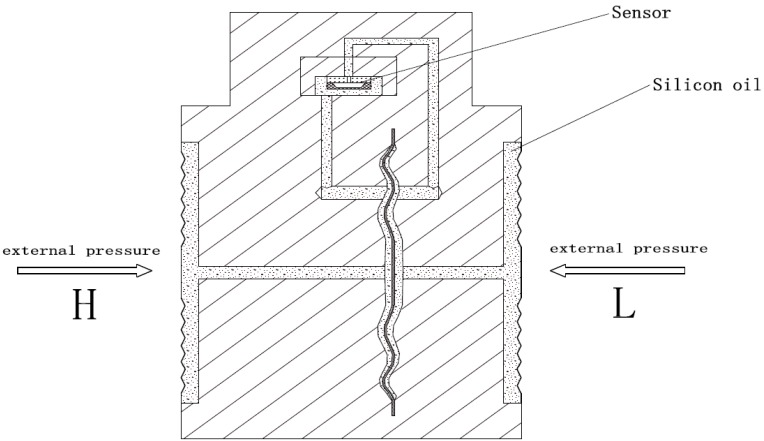
Schematic of the packaged piezo-resistive differential pressure sensor.

The piezo-resistive pressure sensor used in this research is packaged as shown in Figure 1. The inside of the packaged differential pressure sensor is filled with a low thermal expansion rate silicon oil, which serves as the pressure transmission media. The external pressure is applied on the surface of the isolated steel membrane and transferred to the sensor by the silicon oil. The differential pressure (P_d_) can be calculated by the pressure on the L side (P_L_) and the pressure on the H side (P_H_) of the packaged sensor, which is P_d_ = P_H_ − P_L_.

### 2.1. Temperature Effect

**Figure 2:**
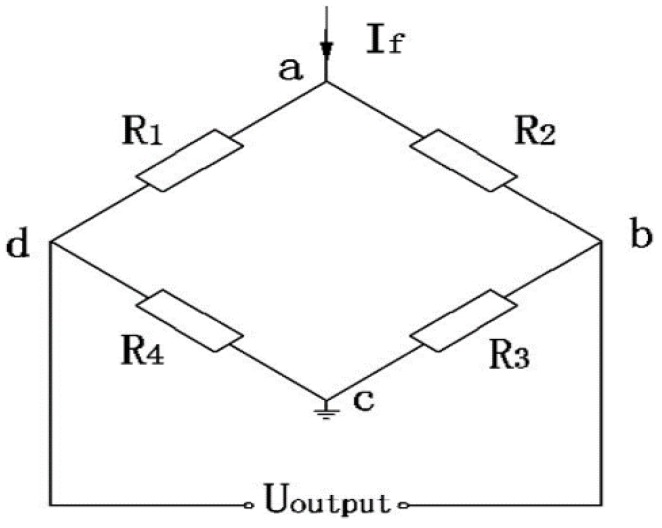
Wheatstone bridge.

The configuration of the piezo-resistive pressure sensor is a symmetrical Wheatstone bridge (R_1_ = R_2_ = R_3_ = R_4_ = R), which is illustrated in Figure 2. By analyzing this Wheatstone bridge the output voltage can be expressed as follows [27]:

(1) $$U_{output}=I_{f}R_{0}\pi_{0}\left[1+(\alpha +\beta +\alpha \beta \Delta T)\Delta T\right]\left[\sigma_{o}+\frac{\alpha_{s}-\alpha_{g}E_{0}\Delta T}{1+\mu }+\theta \right]$$

where $I_{f}$ is a constant current supplied by a steady current source, $R_{0}$ and $\pi_{0}$ are the resistance value and piezo-resistive coefficient at room temperature. The contents of the first bracket represent the temperature influence, where α and β are the temperature coefficients of R and π, respectively, ∆T is the change in the environmental temperature value. Three items in the second bracket stand for the total force applied to the sensor, where the first item $\sigma_{o}$ is the original stress applied on the sensor, the second item characterizes the additional stress when the ambient temperature varies, $\alpha_{s}$ and $\alpha_{g}$ are the thermal expansion coefficients of silicon and glass, respectively, and $E_{0}$ is the temperature coefficient of silicon at the Kelvin temperature, μ devotes the Poisson ratio of the monocrystalline silicon, the third item θ is an offset item that illustrates the fabrication inconsistencies between every bridge arm resistance.

It can be observed from Equation (1) that the actual bridge resistance and piezo-resistive coefficient directly depend on the environmental temperature. Furthermore, the thermal expansion discrepancy between the sensor cup and the glass base becomes non-ignorable as the environmental temperature varies a lot. The relationship clearly unveils the essence of the nonlinearity characteristic between input pressure and output voltage when the environmental temperature changes.

### 2.2. Static Pressure Effect

**Figure 3:**
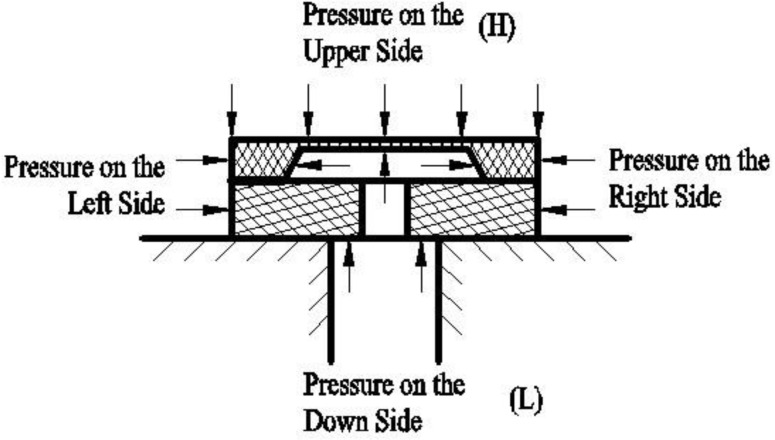
The cross section of the differential pressure sensor.

The silicon cup is glued on the glass base, the cross section of which is shown in Figure 3. The the H side of the silicon cup is set as the reference side and the pressure acting on this side is called the static pressure. It can be seen that the pressure in the horizental direction can be offset by the structural symmetry of the silicon cup. Nevertheless, the pressure in the vertical side would cause the deformation of the silicon cup which means a change of the Wheatstone bridge’s arm resistance. Then the output of the sensor is determined according to the Wheatstone bridge state. There are two main aspects that can be taken as the reasons that cause the static pressure effect. On the one hand, there are distinct differences between the mechanical characteristics of monocrystalline silicon and glass. On the other hand, the overall structure formed by the silicon cup and the glass base is not symmetrical. Considering the factors mentioned above, the silicon cup and the glass base may deform quite differently from each other under the same pressure conditions, which will result in a nonlinear output of the sensor. Generally, the static pressure is higher than the differential pressure in actual situations. Moreover, the more the pressure applied on the packaged pressure sensor the more severely the mismatch deformation between the silicon cup and the glass base is. High static pressure may force the sensor working out of the ideal working pressure range. Obviously, to maintain the same high measurement precision as in the condition without static pressure, it is necessary to perform synthetic compensation for occasions of high static pressure.

## 3. Kernel Extreme Learning Machine

As a kind of single layer feedforward network, its structure simplification and computational efficiency endow the ELM with the ability to sufficiently approximate the actual mapping structure embedded in the sample at an extremely fast speed. Suppose an independent and identical distributed sample including N data points as (*x_1_, y_1_*), (*x_i_, y_i_*), (*x_N_, y_N_*) ∈ ***R****^n^* × ***R*** where *x_i_* is input, *y_i_* is output. The details of the approximation function can be expressed as:

(2) $$\{\begin{array}{c} \sum_{j=1}^{L}\beta_{1}h\left(\omega_{1}x_{1}+b_{1}\right)=y_{1},\\ \cdots \\ \overset{L}{\underset{j=1}{\sum }}\beta_{j}h\left(\omega_{j}x_{i}+b_{j}\right)=y_{i},\\ \cdots \\ \sum_{j=1}^{L}\beta_{L}h\left(\omega_{L}x_{N}+b_{L}\right)=y_{N}. \end{array}$$

where *L* is the number of the hidden layer, $\beta_{j}$ represents the output layer weight links the *j*-th hidden node to the output, $\omega_{j}$ and $b_{j}$ denote the input layer weights and bias of the *j*-th hidden node, respectively, h(·) is the activation function for ELM. Furthermore, Equation (2) can be written in matrix form:

(3) Hβ=Y

where **H** is the hidden layer computation matrix: $H=\left[\begin{array}{c} h(x_{1})\\ \vdots \\ h(x_{2}) \end{array}\right]$, β is the output layer weight vector and Y is the output matrix. Given the randomly generated input layer weights and the specified activation function, the output layer weight vector can be calculated in terms of the norm least square method. Then, the output layer weight vector can be represented as follows:

(4) $$\beta =H^{+}Y$$

where $H^{+}$ is the Moore-Penrose generalized inverse of **H**.

Huang et al. proposed the unified ELM in pursuit of a more unified framework for classification and regression. According to the regularization theory, a positive item 1/C (where C is the penalty parameter) is incorporated into the calculation of the output weight vector and Equation (4) turns into:

(5) $$\beta =H^{T}{\left(HH^{T}+\frac{I}{C}\right)}^{-1}Y$$

Meanwhile, the regression results with respect of inputs can be defined as:

(6) $$f\left(x\right)=HH^{T}{\left(HH^{T}+\frac{I}{C}\right)}^{-1}Y$$

(7) $$HH^{T}=\left[\begin{array}{c} h(x_{1})h(x_{1})\\ \vdots \\ h(x_{i})h(x_{1})\\ \vdots \\ h(x_{N})h(x_{1}) \end{array}\begin{array}{c} \cdots \\ \ddots \\ \cdots \\ \ddots \\ \cdots \end{array}\begin{array}{c} h(x_{1})h(x_{j})\\ \vdots \\ h(x_{i})h(x_{j})\\ \vdots \\ h(x_{N})h(x_{j}) \end{array}\begin{array}{c} \cdots \\ \ddots \\ \cdots \\ \ddots \\ \cdots \end{array}\begin{array}{c} h(x_{1})h(x_{N})\\ \vdots \\ h(x_{i})h(x_{N})\\ \vdots \\ h(x_{N})h(x_{N}) \end{array}\right]$$

In light of the Cover theorem, inputs in a nonlinear separable space become easier to separate by mapping them into a higher dimensional space. The kernel trick is always the first choice for the implementation of this work, the KELM thus uses it to replace the elements in Equation (7). So Equation (7) can be rewritten as:

(8) $$\Omega_{\mathrm{KELM}}=HH^{T}=\left[\begin{array}{c} K(x_{1},x_{1})\\ \vdots \\ K(x_{i},x_{1})\\ \vdots \\ K(x_{N},x_{1}) \end{array}\begin{array}{c} \cdots \\ \ddots \\ \cdots \\ \ddots \\ \cdots \end{array}\begin{array}{c} K(x_{1},x_{j})\\ \vdots \\ K(x_{i},x_{j})\\ \vdots \\ K(x_{N},x_{j}) \end{array}\begin{array}{c} \cdots \\ \ddots \\ \cdots \\ \ddots \\ \cdots \end{array}\begin{array}{c} K(x_{1},x_{N})\\ \vdots \\ K(x_{i},x_{N})\\ \vdots \\ K(x_{N},x_{N}) \end{array}\right]$$

Finally, the KELM regression model can be denoted as:

(9) $$f\left(x\right)={\left[\begin{array}{c} K(x,x_{1})\\ \vdots \\ K(x,x_{j})\\ \vdots \\ K(x,x_{N}) \end{array}\right]}^{T}{\left(\Omega_{\mathrm{KELM}}+\frac{I}{C}\right)}^{-1}Y$$

Since the kernel function is one of the critical factors that contribute to approximation capability, a reasonable choice of kernel function is necessary. Actually, kernel that meets the Mercer kernel condition, namely admissible kernel, can be set as a kernel function. A typical and extensively used kernel function is the Radial Basis Function (RBF) kernel, which is defined as [28]:

(10) $$K_{RBF}=exp\left(-\frac{{∥x-x_{i}∥}^{2}}{2\sigma^{2}}\right)$$

where the spread σ is the only dependent kernel parameter to be selected. However, the proposed scheme is also feasible with other kernels in a same manner.

## 4. Coupled Simulated Annealing and Simplex Search

### 4.1. Coupled Simulated Annealing (CSA)

Noting that the setting of parameters in KELM is vital to the final model performance, the coupled simulated annealing (CSA) optimization method proposed by Xavier de Souza et al. [29] is employed to perform the parameter search. Like a previously published algorithm, simulated annealing [30] simulates the metal annealing process. According to statistical mechanics, the distribution of atom energy in the *i*-th state at a specified temperature level conforms to the Boltzmann formulation:

(11) $$P(E=E_{i})=\frac{1}{Z}\exp(-\frac{E_{i}}{k_{B}})$$

where $E_{i}$ is the energy in the *i*-th state, $k_{B}$ is the Boltzmann constant, *Z* is the partition function. The simulated annealing is a kind of heuristic algorithm that iterates in the decreasing direction of object function. The Metropolis principle also allows the iteration process to accept the increasing object function value with a relative small probability which contributes to the dynamics to jump out of the local minimum. The coupled simulated annealing is developed from the classical simulated annealing but the parallel mechanism within it is distinct from the latter. The coupled simulated annealing introduced a multi-start initialization into the probing process over the solution space to supply more a priori information. The coupled item is created to help decide if the less favorable solution is accepted and to simplify the computational complexity. The coupled item and acceptance probability take the following form:

(12) $$A_{\Theta }\left(\gamma ;x_{i}\to y_{i}\right)=\frac{\exp(\frac{E(x_{i})}{T_{k}^{acc}})}{\gamma }$$

(13) $$\gamma =\underset{x_{i}\in \Theta }{\sum }\exp(\frac{E(x_{i})}{T_{k}^{acc}})$$

where $x_{i}$ and $y_{i}$ are states belonging to the possible state space, $E(x_{i})$ denotes the energy in $x_{i}$, $T_{k}^{acc}$ is the acceptance temperature at the *k*-th iteration, Θ represents the current state set, $A_{\Theta }\left(\gamma ;x_{i}\to y_{i}\right)$ illustrates the acceptance probability from state $x_{i}$ to state $y_{i}$ with the coupled item γ. The coupled simulated annealing algorithm can be depicted as follows:

(1) Initialization: M random solutions are assigned to Θ. Evaluate the energy $E(x_{i})$ and coupled term γ. Set $T_{k}$ as $T_{0}$, $T_{k}^{acc}$ as $T_{0}^{acc}$ and the iteration index k equals to zero. The variance of the acceptance probability is calculated according to $\sigma_{D}^{2}=0.99(m-1/m^{2})$, where *m* is the number of all states included in Θ. The rate controls the temperature variation marked as α is set to 0.05.
(2) A new state $y_{i}$ is generated that corresponds to the current state $x_{i}$ in the state space by $y_{i}=x_{i}+\varepsilon_{i}$ where $\varepsilon_{i}$ is independently and randomly sampled from a normal distribution at temperature $T_{k}$ as $g(\varepsilon_{i},T_{k})$. Evaluate all the energy values $E(y_{i})$*,* ∀i=1,⋯,m.
(3) The new state is accepted if $E\left(y_{i}\right)$ ≤ $E(x_{i})$ or a random number *rand* is generated by a uniform distribution in [0, 1] and compared with the acceptance probability $A_{\Theta }\left(\gamma ;x_{i}\to y_{i}\right)$ obtained by Equation (12) when it satisfies the condition $A_{\Theta }>rand$. Otherwise, the old state remains. Assess γ and return to step 2 to achieve thermal equilibrium condition.
(4) Adjust the acceptance temperature $T_{k}^{acc}$ by the rules: if $\sigma^{2}<\sigma_{D}^{2},T_{k}^{acc}=\left(1-\alpha \right)\times T_{k-1}^{acc}$, if $\sigma^{2}>\sigma_{D}^{2},T_{k}^{acc}=(1+\alpha )\times T_{k-1}^{acc}$.
(5) Increment the annealing time *k* and decrease the temperature according to the annealing scheme: $T_{k}=T_{0}/k$.
(6) Terminate the iteration if the current energy value meets the stopping criterion, otherwise, go back to step 2.

### 4.2. Simplex Search

The simplex search was developed by Nelder and Mead [31] as a simplex is a geometric figure that has *n* + 1 vertices. The expansion operation and the contraction operation along the line of reflection allow the simplex search to considerably reduce the computation time. Assuming the initial simplex is $X=\{x_{1},x_{2},\cdots ,x_{n+1}\}$ so that the output is assigned as *X* because it is our new simplex. It should be noted that *X* is a *n* × (*n* + 1) matrix, where each column represents a simplex vertex. The simplex searching process is described as follows:

1. Build *n* + 1 vertices of *X*, evaluate and sort their function values.
2. Compute the reflection point $x_{r}$ by $x_{r}=\bar{x}+\alpha \left(\bar{x}-x_{n+1}\right)$, where $\bar{x}=\frac{1}{n}\sum_{i=1}^{n}x_{i}$ is the centroid of the *n* points except for the worst point as $x_{n+1}$. If $f\left(x_{1}\right)\leq f(x_{r})\leq f(x_{n+1})$, accept the reflected point $x_{r}$.
3. If $f(x_{r})<f(x_{1})$, perform the expansion operation as $x_{e}=\bar{x}+\beta \left(x_{r}-\bar{x}\right)$. If $f(x_{e})\leq f(x_{r})$, accept the expanded point $x_{e}$; otherwise accept $x_{r}$.
4. If $f(x_{r})\geq f(x_{n})$, a contraction should be done utilizing $\bar{x}$ with the better point of $x_{n+1}$ and $x_{r}$:
  (a) If $f(x_{r})<f(x_{n+1})$, then outside contraction: $x_{c}=\bar{x}+\gamma (x_{r}-\bar{x})$. If $f(x_{c})<f(x_{r})$, accept $x_{c}$; otherwise go to step 5.
  (b) If $f(x_{r})\geq f(x_{n+1})$, then inside contraction: $x_{c}=\bar{x}-\gamma (\bar{x}-x_{n+1})$. If $f(x_{c})\leq f(x_{n+1})$, accept $x_{c}$; otherwise go to step 5.
5. Calculate *f* at the *n* points $v_{i}=x_{1}+\delta \left(x_{i}-x_{1}\right),i=2,\cdots ,n+1.$ The vertices at the next iteration are made up of $X=\{x_{1},v_{2},\cdots ,v_{n+1}\}$.

**Figure 4:**
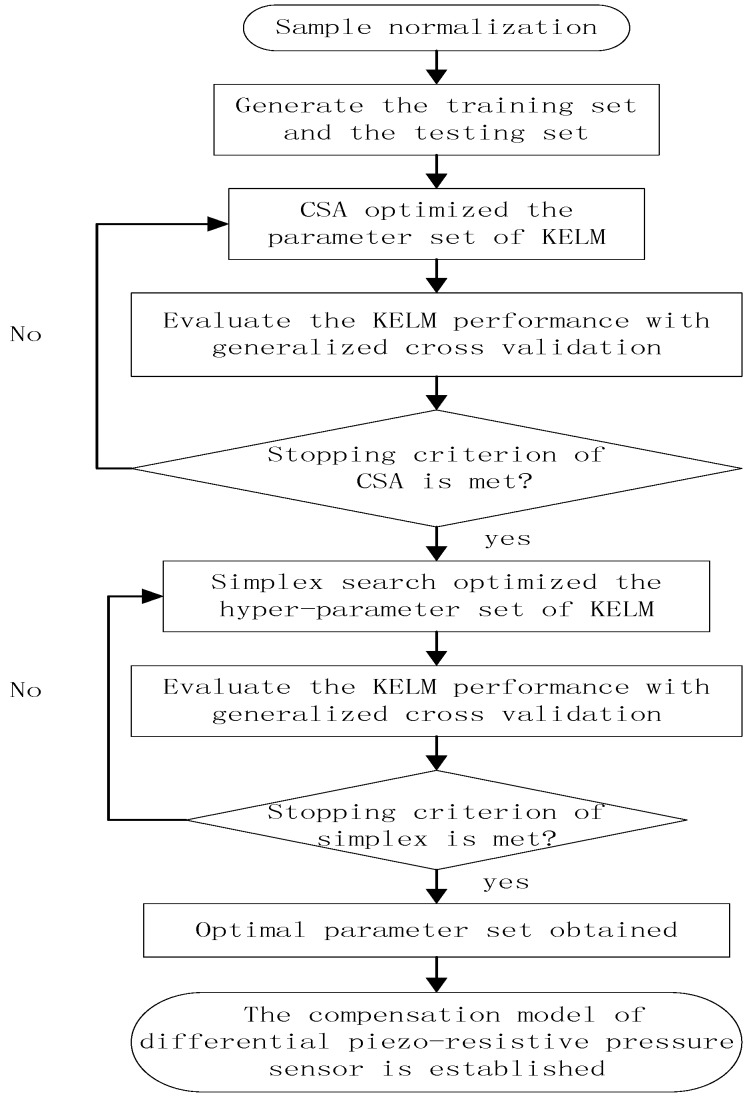
The flowchart of the coupled simulated annealing (CSA) and simplex optimized KELM compensation model.

The flowchart of the temperature and synthetic compensation algorithm is illustrated in Figure 4.

Taking all the merits of CSA and simplex search into account it is reasonable to model the non-linear relationship between the input and output of the piezo-resistive differential pressure sensor as follows:

(1) Divide the normalized sample into training set and testing set according to the engineering requirements.
(2) Initialize the KELM with a random parameter set in an acceptable range.
(3) Initialize the parameters in CSA such as the number of states at a certain temperature M, the coupled term value γ, the starting temperature $T_{0}$, the starting acceptance temperature $T_{0}^{acc}$ and the temperature step regulating rate α.
(4) Evaluate the function iteratively until the maximum iteration number is reached or the fitness is less than the limit $\varepsilon_{1}$. A suboptimal hyper-parameter set $\left(C_{1},\sigma_{1}\right)$ is found through this step.
(5) The simplex search is performed with the start solution as $\left(C_{1},\sigma_{1}\right)$ until the maximum iteration number is reached or the difference between fitness in two successions is small than the limit $\varepsilon_{2}$. Finally, a more satisfactory parameter set as $\left(C_{2},\sigma_{2}\right)$ can be obtained.

## 5. Experiments and Result Analysis

### 5.1. Calibration Experiment Setup

A calibration experiment was performed on a 0.065% grade pressure sensor without compensation [32] to provide data for modeling the KELM approach. The measurement range of the pressure sensor used in this calibration is from −1000 kPa to 1000 kPa, and the working temperature range is from −20 °C to 70 °C. The environmental temperature and the differential pressure output, which were marked as T_AD_ and U_AD_, are converted by an analog-digital (A/D) converter. A two channel pressure controller exerts the H side pressure (P_H_) and the L side pressure (P_L_).

**Figure 5:**
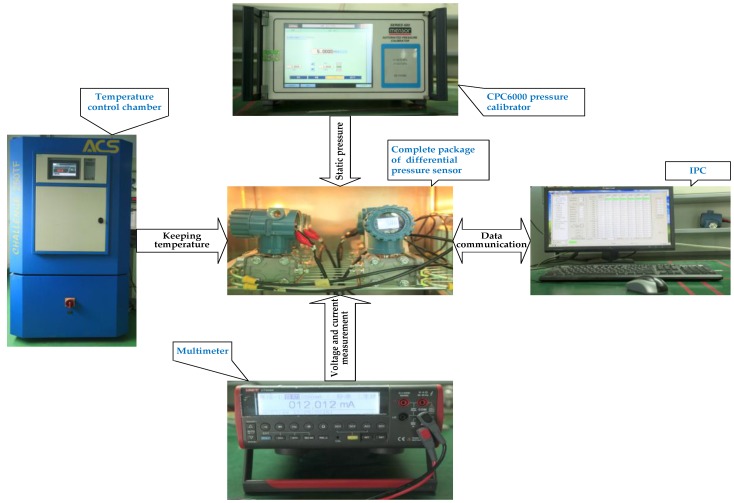
Setup for the calibration experiments.

The static pressure (P_H_) which is denoted by *P_s_* is sampled at 14 levels (0, 1000, 2000, 2500, 3000, 4000, 4500, 5000, 6000, 6500, 7000, 8000, 8500 and 9000 kPa). The differential pressure denoted as DP is specified in a step of 125 kPa from −1000 kPa to 1000 kPa at each static pressure level. Data calibration is performed for five temperature preservation processes at temperatures of −20, 0, 20, 50 and 70 °C. Every temperature preservation process takes about 3 h. The input variables used to construct the KELM include the differential pressure output U_AD_, the static pressure *SP* and the environmental temperature T_AD_, while the output is the differential pressure *DP*. A total of 17 × 14 × 5 = 1190 input-output pairs were collected when the experiments finished. The experimental setup is shown in Figure 5.

**Figure 6:**
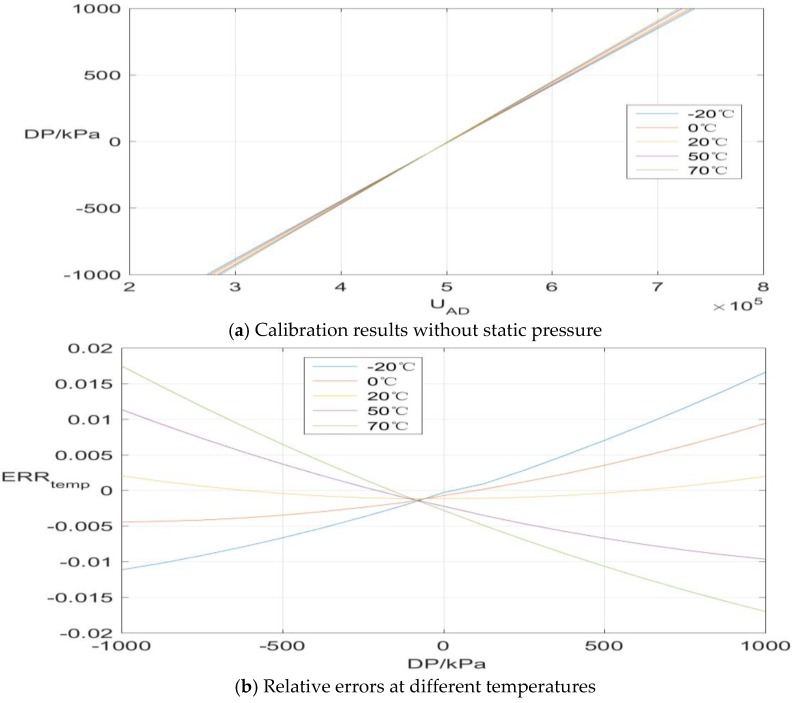
Pressure sensor’s output and relative error at different temperatures.

**Figure 7:**
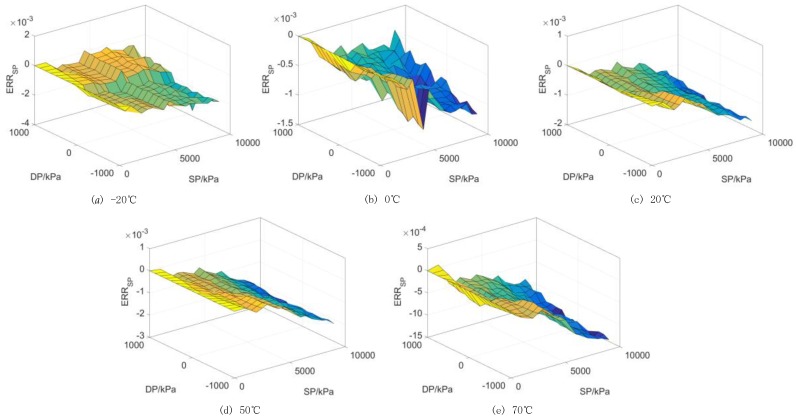
Static pressure errors of the pressure sensor. (**a**) Static errors at −20 °C; (**b**) Static errors at −0 °C; (**c**) Static errors at 20 °C; (**d**) Static errors at 50 °C; (**e**) Static errors at 70 °C.

The calibration data results without static pressure (*SP* = 0 kPa) are illustrated as Figure 6a. It can be seen in Figure 6a that the sensor’s output performance is affected by the environmental temperature notably without any static pressure participation. To be more specific, relative errors (Err_temp_) with respect to the linear fit characteristic according to data at 20 °C are depicted in Figure 6b, which shows the maximum drift value reaches 1.75%. On the other hand, the maximum measurement errors caused by the static pressure effect are 0.31%, 0.11%, 0.16%, 0.2% and 0.13% corresponding to temperature settings of −20, 0, 20, 50 and 70 °C, respectively. As demonstrated in Figure 7, the measurement errors increase gradually with the static pressure and also fluctuate randomly at different static pressure levels. As a matter of fact, both the silicon cup and the glass base experience distortion to a different degree when the force status and temperature conditions change, which might be the main factor contributing to this phenomenon. To meet the high engineering requirements, a synthetic compensation strategy should be taken into account to address the remarkable temperature and static pressure effects.

### 5.2. Logarithmic Transformation of Dependent Parameters and Normalization

To speed up and facilitate the search process in the parameter space, the penalty parameter C and the kernel parameter σ use l n to accomplish a logarithmic transformation. Additionally, features in the calibration experiment have mismatched levels of magnitude, which would result in an undesirable solution. Thus, the experiment sample should be normalized into the [−1, 1] space according to the form:

(14) $${\hat{x}}_{i}=\frac{x_{i}-(x_{i}(\max)-x_{i}(\min))/2}{(x_{i}(\max)-x_{i}(\min))/2},$$

where $x_{i}$ is the i-th variable of a data point, and $x_{i}(\max)$ and $x_{i}(\min)$ are the upper limit and lower limit of i-th variable, respectively. After normalizing the experimental data, a training set and a testing set should be partitioned before modeling.

### 5.3. Compensation Result Analysis

To evaluate the compensation effectiveness of the CSA and simplex search optimized KELM (CSA-simplex-KELM) model, several other methods, including BP, RBF, particle swarm optimization optimized SVM (PSO-SVM), particle swarm optimization optimized LSSVM (PSO-LSSVM) and ELM were also investigated. The performance measure of all the compensation algorithms is the absolute relative error, which takes the form of:

(15) $$Err_{i}=\left|\frac{DP_{i}^{\prime }-DP_{i}}{DP_{FS}}\right|\times 100\%,$$

where *i* is the index of differential pressure, $DP^{\prime }$ is the compensated differential pressure, DP is the reference differential pressure, and $DP_{FS}$ is the sensor full scale.

Because PSO and CSA are heuristic algorithms, the parameter set (C,σ) is defined as an individual solution in their search space. To achieve a balance between the regression accuracy and algorithmic stability, the mean squared error (MSE) of *Err* is taken as the algorithm fitness function. The parameters of each method are tabulated in Table 1. Considering the importance of cross validation for the determination of the model and the computation efficiency, generalized cross validation is adopted to prevent overfitting [33]. The whole coding is implemented with the help of LS-SVMlab toolbox and libsvm toolbox on the MATLAB (2015b) platform (The Mathworks, Inc., Natick, MA, USA) [34,35].

The differential pressure sensor can work in two ways: with or without static pressure. In the first case the static pressure effect is neglected, which simplifies the task to a temperature compensation problem. From a more practical viewpoint, the second case is more complicated as the temperature and static pressure are coupled. These two cases are discussed in the following subsections.

#### 5.3.1. Temperature Compensation

A training set consists of nine differential pressure levels is uniformly selected from −1000 kPa to 1000 kPa in the working temperature range, which means 45 data pairs in all. The rest of the data without static pressure are put into the testing set. The number of hidden layer nodes except for SVM and LSSVM are determined as in Table 2.

Four performance indices—minimum (min), maximum (max), mean (mean), variance (var) of the averaged training set errors (Err)—are taken to evaluate the compensation performance of every model, and the details are summarized in Table 3 and Table 4. Some conclusions can be directly inferred from the observation of the training set errors in Table 3 and testing set errors in Table 4. The BP neural network does not perform as well as other models in both the training set and testing set, even with the simplest structure, which means it falls into a local optimal solution. The RBF neural network is among the best when merely considering the testing set results, but it may lose some features during the training period, which makee the maximum error comes to 3.8180 × 10^−4^.

Because the PSO-LSSVM model makes use of all the training data to learn the nature of the mapping relationship, it is not only superior to PSO-LSSVM in all indices, but also gives a rather similar result compared with the ELM. Obviously, the models within the ELM framework can provide a more desirable improvement of measurement accuracy and stability than others.

**Figure 8:**
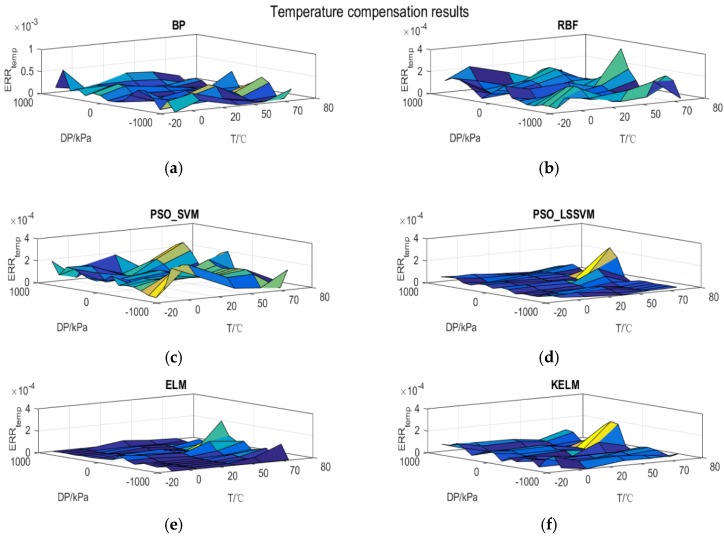
Temperature compensation results obtained by different models. (**a**) BP temperature compensation results; (**b**) RBF temperature compensation results; (**c**) PSO-SVM temperature compensation results; (**d**) PSO-LSSVM temperature compensation results; (**e**) ELM temperature compensation results; (**f**) KELM temperature compensation results.

As seen in the compensation results of all the models, shown in Figure 8, the maximum compensation error occurs at almost 70 °C which explains why the temperature effect is vital to the pressure measurement and difficult to eliminate completely. There is no tendency in any of the compensation results of all the models, which confirms the validity of these models. The PSO-LSSVM, the ELM and the KELM share almost the same global compensation performance, while the proposed KLEM model requires the least number of hidden layer nodes. From the engineering viewpoint, hence, the KELM presents the most satisfactory comprehensive performance in controlling compensation error and convenience of implementation.

#### 5.3.2. Synthetic Compensation

If we note that sensors always work under the influence of the static pressure in most practical environments, a synthetic compensation strategy is critical. Within the working temperature range (−20 °C–70 °C), nine differential pressure levels were uniformly selected from −1000 to 1000 kPa and seven static pressure levels (1000, 2500, 4000, 5000, 6500, 8000 and 9000 kPa) make up the training set for the modeling process, which means we have 315 data points. The rest of the calibration data forms the testing set. Except for the PSO-SVM and the PSO-LSSVM, the other models’ performance is related to their topology structure, so the number of hidden layer nodes except for SVM and LSSVM are set as in Table 5.

Like in the temperature compensation, the training set and testing set compensation results are summarized in Table 6 and Table 7, respectively. In comparison with the temperature compensation data, the overall quality of the synthetic compensation is reduced to a certain extent. This change proves that the static effect is really vital to the synthetic performance of the sensor. By analyzing the results in Table 6 and Table 7, several conclusions can be reached: firstly, the maximum training error and testing error of the BP neural network are 8.8874 × 10^−4^ and 1.4886 × 10^−3^. This exposes another defect of BP in that its generalization ability is limited while the training process seems successful; secondly, the RBF network lacks searching ability as the maximum error obtained by it reaches 1.4557 × 10^−3^ in the learning period; thirdly, the ELM approach does not perform as well as in the temperature compensation problem when the sample scale and model complexity increase; fourthly, although the maximum error of the PSO-LSSVM associated with the testing set is the best, the optimization method which is the bottleneck to sufficiently explore the parameter space for the maximum error in the training set reaches 1.2025 × 10^−3^; lastly, in combination with the advantages of regularization theory and kernel trick, the maximum errors of the proposed KELM method in the training set and testing set are 8.2560 × 10^−4^ and 1.0836 × 10^−3^, which demonstrates an ideal synthetic compensation performance at a low computation cost.

**Figure 9:**
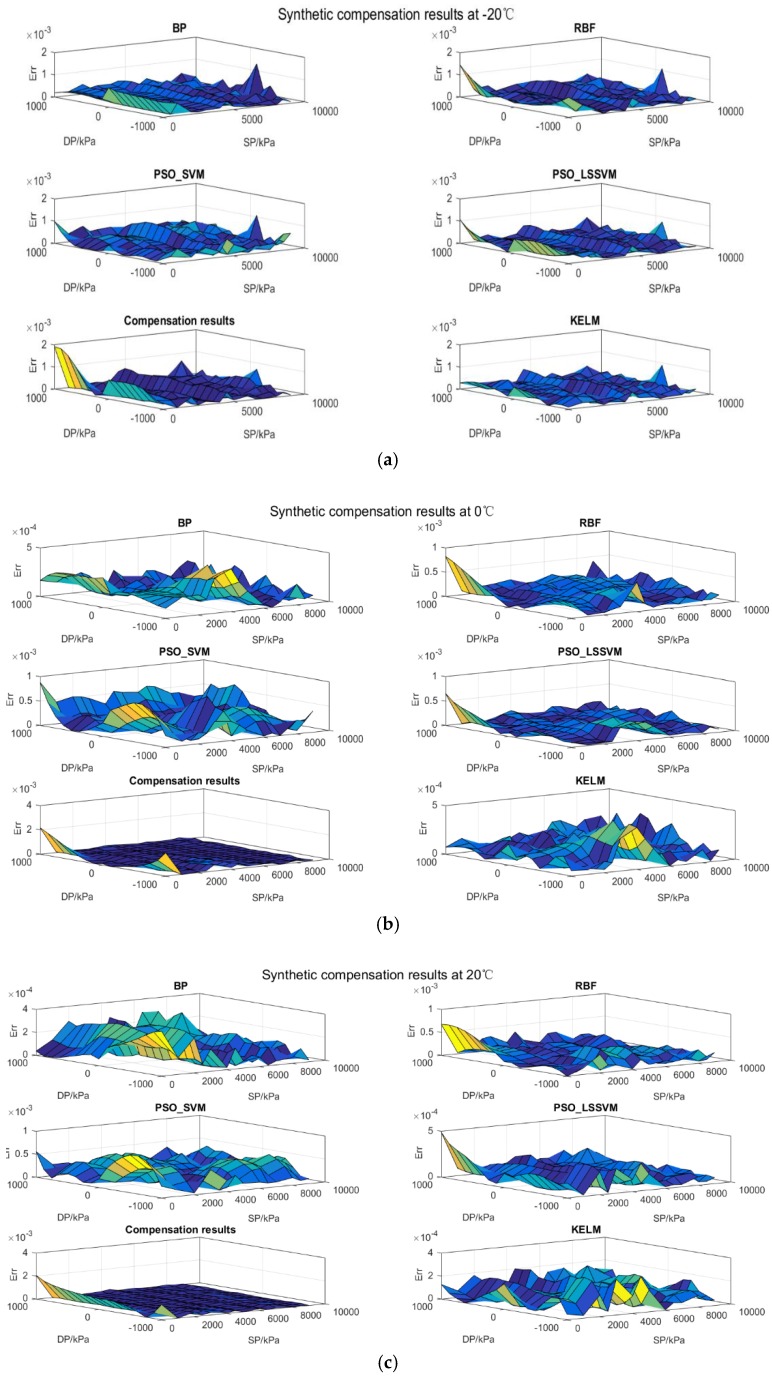

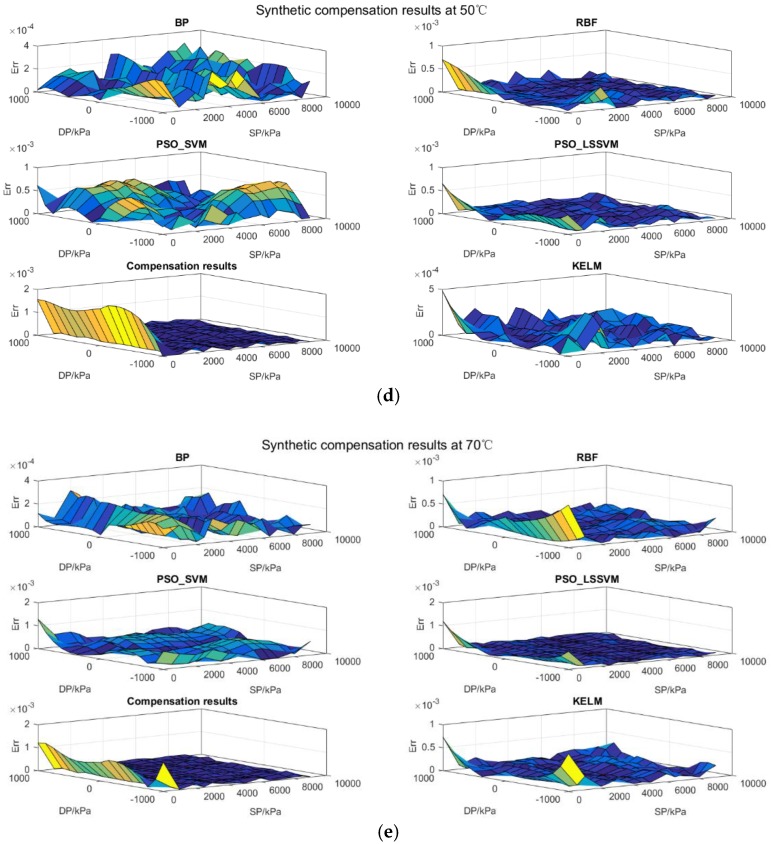
Synthetic compensation results obtained by different models. (**a**) All synthetic compensation results at −20 °C; (**b**) All synthetic compensation results at 0 °C; (**c**) All synthetic compensation results at 20 °C; (**d**) All synthetic compensation results at 50 °C; (**e**) All synthetic compensation results at 70 °C.

All the synthetic compensation details are illustrated in Figure 9, where all the maximum synthetic compensation errors occur at −20 °C except for the ELM, which is due to two facts: the stress between the silicon cup and the glass becomes higher with the decrease of the environmental temperature; the ELM model is not suitable for learning the structure contained in this dataset. Since the training set lacks information about the sensor performance without static effect, the compensation error of RBF and PSO-LSSVM in all temperature states without static pressure is the maximum. It also indicates that the generalization ability of RBF and PSO-LSSVM is not satisfactory for this problem in some way. However, the KELM alleviates both the temperature effect and the static pressure effect and achieves a robust and accurate compensation model which fulfils the engineering requirements [30].

## 6. Conclusions

A synthetic compensation approach that incorporates optimization methods into the framework of KELM is presented in this research. Making full use of regularization theory and kernel trick, the kernel version of the ELM (KELM) is adopted to guarantee the learning and generalization ability of the compensation model. The CSA and simplex search are applied to perform the search of an appropriate parameter set for KELM. The proposed synthetic compensation scheme avoids a complex network topology and speeds up the computation process. The temperature compensation and synthetic compensation results show that it is an effective and efficient compensation method even when dealing with distinct datasets.

## Figures and Tables

**Table 1:** Parameters setting of compensation models.

Parameters	PSO-SVM	PSO-LSSVM	CSA-Simplex-KELM
swarm size/state level	30	30	6
iteration number/annealing time	30	30	30
maximum weight	0.9	0.9	
minimum weight	0.4	0.4	
social factor	[1, 3]	2	
cognitive factor	[1, 3]	2	
thermal equibrium steps			5
initial/acceptance temperature			1
regulation rate			0.1
Penalty parameter (C)	[1, 1 × 10^7^]	[1, 1 × 10^7^]	[1, 1 × 10^7^]
Kernel parameter (σ)	[1 × 10^−3^, 10]	[1 × 10^−3^, 10]	[1 × 10^−3^, 10]
maximum interval tolerance (ε)		[1 × 10^−6^, 1]	

**Table 2:** Model configuration for temperature compensation.

Temperature Compensation Methods	Hidden Layer Node Number and Spread Parameter
BP	8
RBF	37; spread:5.7
ELM	36
CSA-simplex-KELM	10

**Table 3:** Temperature compensation results of the training set.

Temperature Compensation Methods	Err (min)	Err (max)	Err (mean)	Err (variance)
BP	6.1336 × 10^−6^	4.4261 × 10^−4^	1.1508 × 10^−4^	1.3032 × 10^−8^
RBF	3.9938 × 10^−6^	3.8180 × 10^−4^	8.9686 × 10^−5^	5.3639 × 10^−9^
PSO-SVM	6.2186 × 10^−6^	3.0263 × 10^−4^	1.1494 × 10^−4^	5.8987 × 10^−9^
PSO-LSSVM	9.9424 × 10^−7^	2.1566 × 10^−4^	3.4931 × 10^−5^	1.7494 × 10^−9^
ELM	1.0264 × 10^−7^	1.2989 × 10^−4^	2.0954 × 10^−5^	8.1310 × 10^−10^
CSA-simplex-KELM	1.5497 × 10^−6^	2.3419 × 10^−4^	4.5105 × 10^−5^	2.0150 × 10^−9^

**Table 4:** Temperature compensation results of the testing set.

Temperature Compensation Methods	Err (min)	Err (max)	Err (mean)	Err (variance)
BP	7.3618 × 10^−8^	5.3030 × 10^−4^	1.4749 × 10^−4^	1.6054 × 10^−8^
RBF	2.3279 × 10^−8^	2.0774 × 10^−4^	7.0203 × 10^−5^	3.5305 × 10^−9^
PSO-SVM	3.1682 × 10^−6^	2.9965 × 10^−4^	1.1040 × 10^−4^	5.0093 × 10^−9^
PSO-LSSVM	3.4454 × 10^−7^	2.8042 × 10^−4^	3.6780 × 10^−5^	2.1839 × 10^−9^
ELM	8.0991 × 10^−7^	2.5388 × 10^−4^	3.2806 × 10^−5^	2.2638 × 10^−9^
CSA-simplex-KELM	3.5241 × 10^−6^	2.4787 × 10^−4^	5.2075 × 10^−5^	1.8499 × 10^−9^

**Table 5:** Model configuration for synthetic compensation.

Temperature Compensation Methods	Hidden Layer Node Number and Spread Parameter
BP	8
RBF	176; spread:3.7
ELM	154
CSA-simplex-KELM	5

**Table 6:** Synthetic compensation results of training set.

Temperature Compensation Methods	Err (min)	Err (max)	Err (mean)	Err (variance)
BP	7.9857 × 10^−7^	8.8874 × 10^−4^	1.3630 × 10^−4^	1.8544 ×10^−8^
RBF	3.0434 × 10^−7^	1.4557 × 10^−3^	1.7424 × 10^−4^	3.3145 × 10^−8^
PSO-SVM	5.0338 × 10^−7^	1.2871 × 10^−3^	2.9328 × 10^−4^	4.5771 × 10^−8^
PSO-LSSVM	6.2260 × 10^−7^	1.2025 × 10^−3^	1.4617 × 10^−4^	2.6931 × 10^−8^
ELM	7.1578 × 10^−7^	2.1745 × 10^−3^	2.4851 × 10^−4^	1.5724 × 10^−7^
CSA-simplex-KELM	1.4895 × 10^−7^	8.2560 × 10^−4^	1.3599 × 10^−4^	1.5597 ×10^−8^

**Table 7:** Synthetic compensation results of testing set.

Temperature Compensation Methods	Err (min)	Err (max)	Err (mean)	Err (variance)
BP	3.5195 × 10^−7^	1.4886 × 10^−3^	1.2296 × 10^−4^	1.4083 × 10^−8^
RBF	1.9854 × 10^−8^	1.2708 × 10^−3^	1.3248 × 10^−4^	1.6446 × 10^−8^
PSO-SVM	4.7972 × 10^−8^	1.2538 × 10^−3^	2.7009 × 10^−4^	3.5158 × 10^−8^
PSO-LSSVM	1.5621 × 10^−7^	9.6256 × 10^−4^	9.5755 × 10^−5^	1.1373 × 10^−8^
ELM	4.6555 × 10^−9^	1.8777 × 10^−3^	1.1114 × 10^−4^	5.3387 × 10^−8^
CSA-simplex-KELM	3.2846 × 10^−7^	1.0836 × 10^−3^	1.1019 × 10^−4^	1.0429 × 10^−8^

## Supplementary Materials

Supplementary File 1
